# From a Suspicious Liver Mass to Splenosis: A Case Report

**DOI:** 10.7759/cureus.79141

**Published:** 2025-02-17

**Authors:** Francisco Marrana, Catarina Tavares, Antónia Furtado, Tatiana Moreira Marques, Gil Faria

**Affiliations:** 1 General Surgery Department, Unidade Local de Saúde de Matosinhos - Hospital Pedro Hispano, Matosinhos, PRT; 2 Anatomy Department, Faculdade de Medicina da Universidade do Porto, Porto, PRT; 3 Radiology Department, Unidade Local de Saúde de Matosinhos - Hospital Pedro Hispano, Matosinhos, PRT; 4 Pathology Department, Isabel Macedo Pinto (IMP) Diagnostics, Porto, PRT; 5 Surgery Department, Faculdade de Medicina da Universidade do Porto, Porto, PRT

**Keywords:** intrahepatic splenosis, laparoscopic liver surgery, liver mri, rare liver mass, splenosis after splenectomy

## Abstract

Splenosis refers to the uncommon phenomenon of heterotopic autoimplantation of splenic tissue within the abdominopelvic or thoracic cavities, typically observed in patients with a history of splenic trauma or surgery. Intrahepatic splenosis is an even rarer occurrence, with its pathophysiology attributed to the "seeding" of splenic pulp fragments into the liver following splenic injury. Alternatively, a hematogenous spread of splenic cells through the splenic veins may also contribute to hepatic implantation. This condition is often asymptomatic and is usually detected incidentally. The absence of specific radiological characteristics makes its diagnosis particularly challenging, as it can be easily mistaken for other hepatic masses, including adenomas or hepatocellular carcinoma. This case report highlights such a diagnostic challenge.

A 51-year-old male patient was referred for further investigation of a well-circumscribed, homogeneous solid liver lesion, incidentally detected during a routine abdominal ultrasound (US) and initially suspected to be focal nodular hyperplasia (FNH). The patient was asymptomatic, with no palpable abnormalities on physical examination. His past medical history included hepatitis B, systemic arterial hypertension, dyslipidemia, nephrolithiasis, and an urgent splenectomy performed a decade earlier due to traumatic rupture. Laboratory tests revealed a mild elevation in liver transaminases, without signs of cholestasis. Immunologic and microbiological analyses ruled out acute hepatitis B. To further assess the hepatic lesion, abdominal magnetic resonance imaging (MRI) was performed, revealing two nodular formations in segments II and III. The lesion appears homogeneous and isointense compared to the liver parenchyma on both T2- and T1-weighted images, with no signal drop observed on T1 out-of-phase sequences. During dynamic imaging following gadolinium ethoxybenzyl diethylenetriamine pentaacetic acid (Gd-EOB-DTPA) administration, the lesion demonstrates intense homogeneous enhancement in the arterial phase, with no washout observed in the portal phase or the 10-minute equilibrium phase, findings suggestive of β-catenin-mutated adenoma, which carries a risk of malignant transformation. Given this concern, after evaluating the operative risk, surgical intervention was recommended. The patient underwent exploratory laparoscopy, during which both lesions were enucleated with clear margins. The postoperative period was uneventful, and he was discharged the following morning. Histopathological examination confirmed the presence of heterotopic splenic tissue within the liver, with no signs of malignancy. This report underscores the importance of considering intrahepatic splenosis in the differential diagnosis of hepatic nodules, particularly in patients with a history of splenectomy, especially when lesions are located near the liver capsule.

## Introduction

Splenosis is defined as the rare case of heterotopic autoimplantation of splenic tissue in the abdominal, pelvic, or even thoracic cavities being reported in 26%-67% of patients with a history of trauma to or surgery of the spleen [1]. The splenic fragments seed onto the exposed vascularized peritoneal surface, most commonly mesentery, omentum, and peritoneum, receiving blood from the surrounding tissue [2]. Intrahepatic splenosis occurs even less frequently, and the pathophysiology of the spreading of splenic tissue is described by two hypotheses. The first hypothesis postulates splenic trauma as the cause of a "seeding" of splenic pulp fragments in the liver [3]. Alternatively, a hematogenous spread of the splenic cells through the splenic veins could result in hepatic splenosis [4].

Splenosis is a rare condition, usually presenting as an asymptomatic mass incidentally discovered. The lack of typical radiological features makes its diagnosis challenging with non-invasive means and difficult to distinguish from other hepatic masses, such as adenoma or hepatocellular carcinoma [5].

This case was previously presented as a poster at the World Congress of Endoscopic Surgery, 29th International Congress of the European Association of Endoscopic Surgery (EAES) in November 2021.

## Case presentation

A 51-year-old man was referred to our hospital for further evaluation of a well-circumscribed, homogeneous solid liver lesion measuring 33 mm in diameter, located in the surface of segment III, suggestive of focal nodular hyperplasia (FNH), which was discovered incidentally during routine abdominal ultrasound (US). He did not present symptoms, and there were no palpable masses at physical examination.

His medical history included hepatitis B, systemic arterial hypertension, dyslipidemia, nephrolithiasis, and an urgent splenectomy due to traumatic rupture 10 years before.

Routine blood analysis showed a mild elevation of liver transaminases (aspartate transaminase (AST): 54 U/L, alanine transaminase (ALT): 93 U/L) with no associated cholestasis, normal values of hemoglobin, and 450,000 platelets/µL. Immunology serology showed the presence of hepatitis B surface antigen (HBsAg), hepatitis B e antigen (HBeAg), and immunoglobulin G against hepatitis B core (IgGHBc) antibodies and the absence of HBs and HBe. Microbiology also discarded the presence of hepatitis B virus (HBV) viral load.

**Figure 1:**
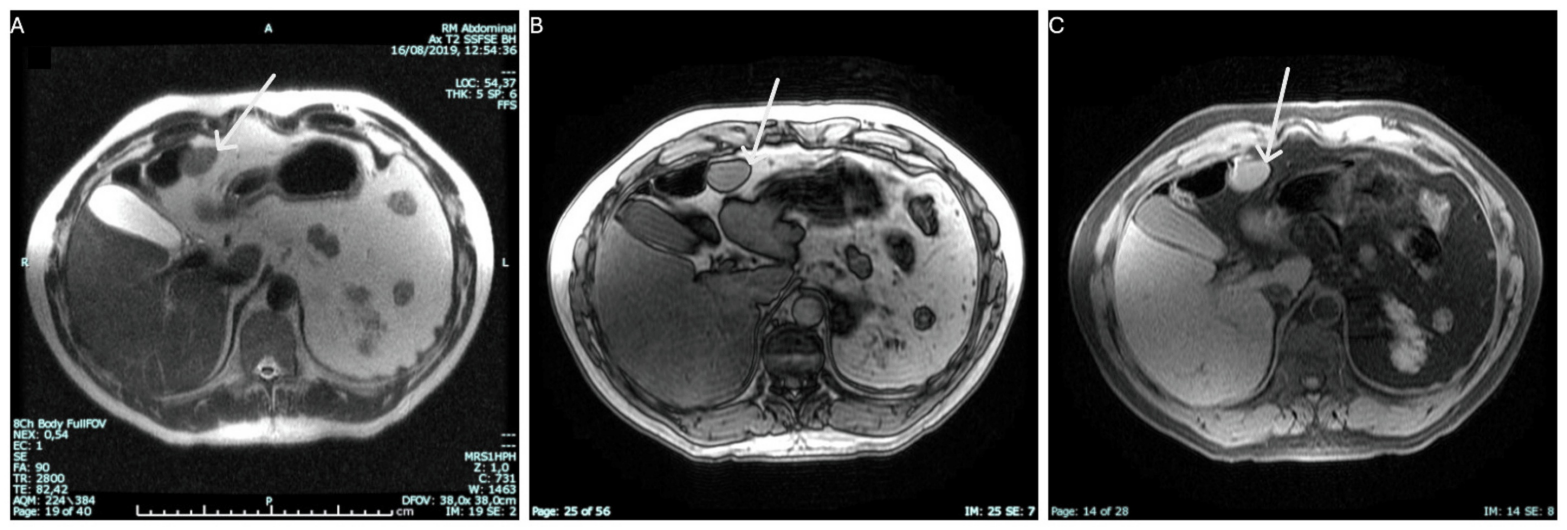
Axial T2 image without fat suppression (A), T1-weighted outphased image, and (B) T1-weighted FatSatFSPGR image(C) The lesion is homogeneous and isointense relative to the liver parenchyma on T2 and T1 weighted images and has no signal drop on T1 outphased images. FatSatFSPGR: fat-saturated fast spoiled gradient-echo

**Figure 2:**
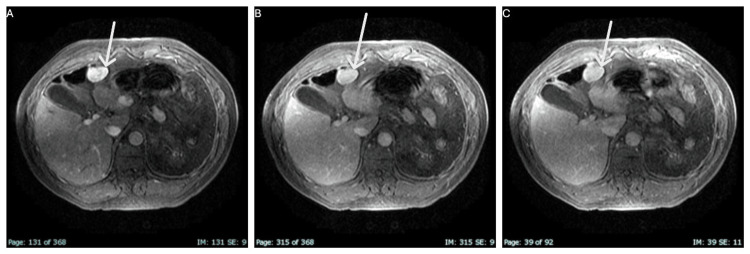
Dynamic evaluation after GdBCM In dynamic evaluation after Gd-EOB-DTPA administration: intense homogeneous enhancement in the arterial phase (A) without washout in the portal phase (B) and 10-minute equilibrium phase (C). Gd-EOB-DTPA: gadolinium ethoxybenzyl diethylenetriamine pentaacetic acid, GdBCM: gadolinium-based contrast media

For better characterization of the hepatic lesion, the patient underwent an abdominal magnetic resonance imaging (MRI). This imaging technique showed an exophytic nodular lesion in the visceral surface of the left hepatic lobe, with 35 mm of greater diameter and another with 22 mm of greater diameter in the surface of segment III. The lesions are homogeneous and isointense relative to the liver parenchyma on T2- and T1-weighted images and have no signal drop on T1 outphased images. There were no retroperitoneal adenopathies identified (Figure 1 and Figure 2).

The imaging pattern of the lesions was potentially worrisome for the risk of malignant transformation of this kind of adenoma. We therefore opted for surgical intervention after clinical evaluations of operative risk, which was accepted and consented to by the patient.

**Figure 3:**
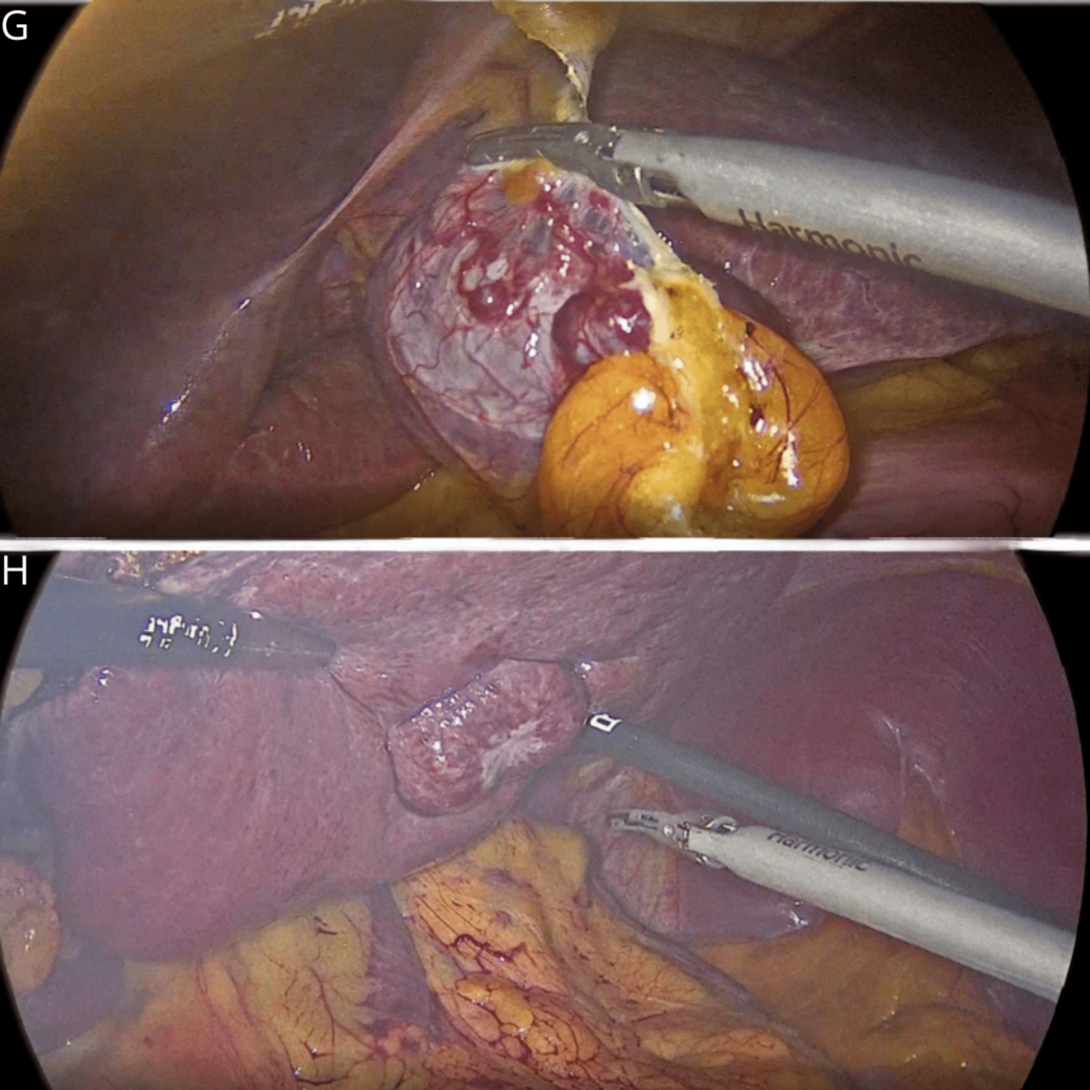
Exploratory laparoscopy Excision of hypervascular lesions in liver segments II (G) and III (H).

An exploratory laparoscopy was performed. We identified both lesions on the surface of segments II and III, with a clear margin from the liver parenchyma, and proceeded with laparoscopic enucleation. The operative time was 50 minutes, and the estimated blood loss was 10 cc (Figure 3).

**Figure 4:**
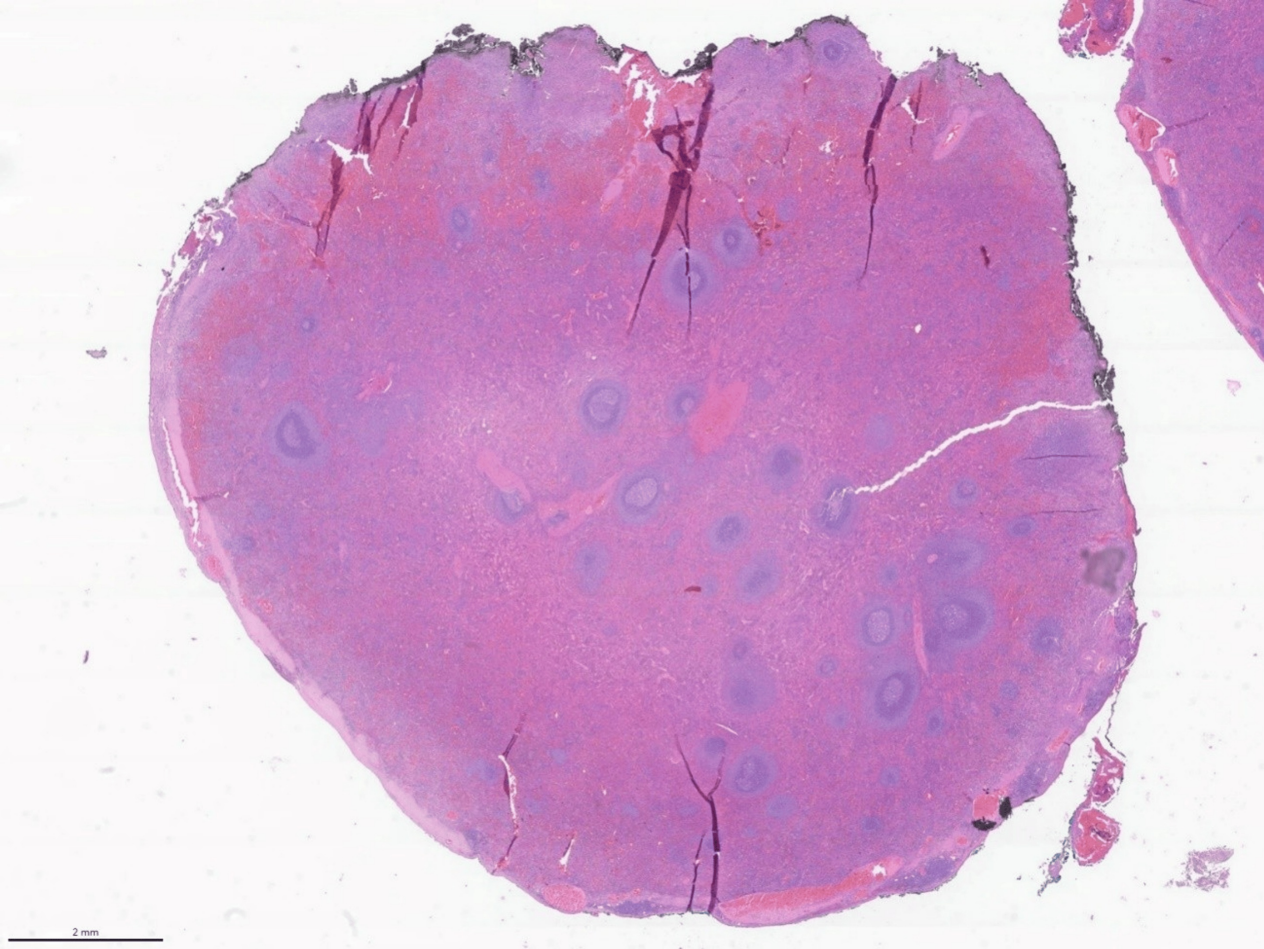
Low-power histological view of splenosis H&E image at low-power magnification showing the entire nodule that corresponds to splenic tissue. The nodule was partially covered by a capsule. There was no representation of liver tissue. H&E: hematoxylin and eosin

**Figure 5:**
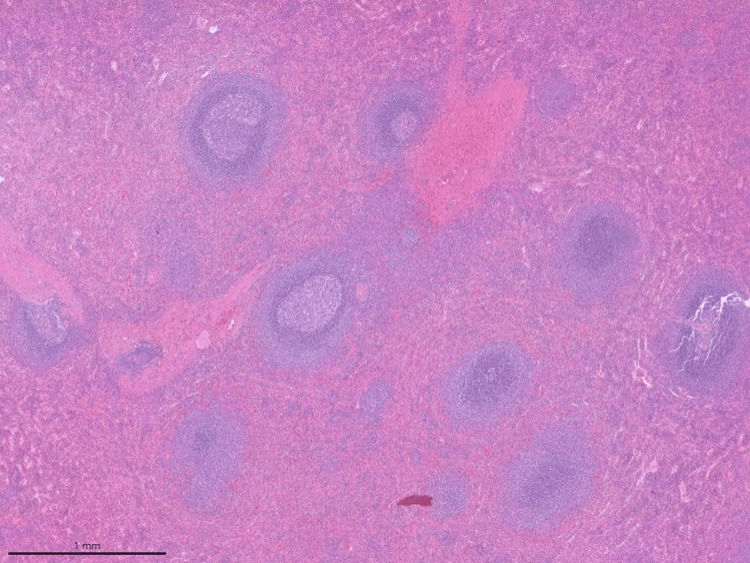
Intermediate-power histological view of splenic tissue H&E image at intermediate-power magnification showing splenic tissue with representation of white pulp and red pulp, without significant architectural changes. H&E: hematoxylin and eosin

There were no postoperative complications, and the patient was discharged the following morning. Histopathology revealed intrahepatic heterotopic splenic parenchyma, with no evidence of neoplasia in either of the two lesions, the measures of which were 3.8 × 2.4 × 1.9 cm and 4.5 × 2.5 cm (Figure 4 and Figure 5).

The follow-up after three months presented no adverse or unanticipated event.

## Discussion

Albrecht reported the first case of splenosis in 1896, but it was not until 1939 when Buchbinder and Lipkoff [5] started to call it as such [6]. According to Luo et al., 41 cases of hepatic splenosis have been published between 1993 and 2016, which illustrates its low prevalence [6]. Hence, knowledge of the clinical features and diagnosis of splenosis remains limited. Emergent laparotomy after trauma was the most frequent reason for splenectomy (88%) [7].

No specific symptoms have been reported in hepatic splenosis patients, although abdominal pain may be present due to heterotopic splenic infarction or compression, resulting in missed diagnosis. Gastrointestinal bleeding and bowel obstruction associated with compression or sudden torsion of the solid lesion were also described. Despite that, most cases remain asymptomatic and are referred because of incidental findings [8,9].

The radiological features of the normal spleen include hyperintensity opposing the liver tissue on T2-weighted images, but similar signal intensity to any residual splenic tissue is the most important feature in cases of splenosis [10]. Nevertheless, all these features are non-specific, which makes reaching an accurate diagnosis with common imaging techniques, such as US, CT, and MRI, an even more arduous task. As a result, intrahepatic splenosis can be misdiagnosed as hepatic adenoma, atypical hepatocellular carcinoma, atypical hemangioma, or metastases, leading to unnecessary surgery or other invasive treatments [8,11]. In reality, patients who present intrahepatic splenosis as a final diagnosis were often previously treated with invasive procedures, including biopsy and surgical resection. Nevertheless, intrahepatic splenosis can replace part of the immunologic function of the removed spleen, which can be beneficial in patients who have undergone splenectomy [12]. Thus, most asymptomatic patients require no treatment at all, with some special situations such as Felty syndrome or idiopathic thrombocytopenic purpura the exceptions to this recommendation [13].

Some existing procedures such as scintigraphy with sensitive technetium-99 m-labeled heat-denatured red blood cells (Tc-99 m-DRBC) are reported to have more specificity and efficiency in diagnosing splenosis, although it does not localize the lesions in an anatomical feature [14,15].

Although with lower sensitivity, scintigraphy with sulfur colloid also proved useful in diagnosing splenosis. Superparamagnetic iron oxide (SPIO) contrast magnetic resonance imaging may also be important for the diagnosis of splenosis, in particular by providing a reliant distinguishing feature from hepatocellular carcinoma (HCC). As reported, intrahepatic splenosis will remain hyperintense relative to the liver parenchyma, while HCC will become hypointense after the SPIO administration [16].

The limitation of this case is not having considered splenosis as a possible diagnosis before operation, due to the radiological suggestion of adenomas with worrisome features.

In this singular case, the past medical history of splenic trauma followed by splenectomy could have raised suspicion of intrahepatic splenosis. Nonetheless, practically any non-invasive diagnostic method could have safely avoided surgery.

## Conclusions

Through this report, we emphasize the importance of considering intrahepatic splenosis as a remote possibility in patients with hepatic nodules who have a history of splenectomy, particularly when its presentation is near the live capsule.
